# A new species of *Margaromantis* Piza, 1982 (Insecta: Mantodea) from Brazil

**DOI:** 10.3897/BDJ.3.e4343

**Published:** 2015-02-03

**Authors:** Eliomar da Cruz Menezes, Freddy Bravo

**Affiliations:** † Universidade Estadual de Feira de Santana, Feira de Santana, Brazil

**Keywords:** Chapada Diamantina, Dictyoptera, Mantidae, Neotropical region, Photinainae, taxonomy.

## Abstract

A second species of the Neotropical mantid genus *Margaromantis* Piza, 1982, *Margaromantis nigrolineata* sp. n. is described from Bahia, Brazil. This new species can be recognized by the presence of a transverse black strip between compound eyes in the vertex; fore femora exhibiting black calluses on the inner face; lacking yellowish strips over the transverse veins on the metathoracic wings; left dorsal phallomere with rectangular ventral lamina, elongated and grooved lateral process, and a flattened, but not twisted apical process that is upwardly recurved.

## Introduction

The genus *Margaromantis* was described by Piza (1982) in monotypy for *Margaromantis margaritaria* Piza, 1982. After several nomenclatural changes this species is currently know as *Margaromantis planicephala* (Rehn, 1916) (Rivera 2010).

Rehn (1916) described the species *Metriomantis planicephala* Rehn which was transferred by Lombardo (1999) to a new monotypic genus *Rehniella*. In independent publications in the same year, Koçak and Kemal (2008) and Özdikmen (2008) proposed new names for *Rehniella*, due to synonymy with *Rehniella* Herbard, 1928 (Orthoptera): in May Koçak and Kemal (2008) proposed the name *Colombiella* and in June Özdikmen (2008) proposed the name *Lombardoa*. In October of the same year, after taking notice of the name proposed by Özdikmen (2008), Koçak (2008) transformed the name *Lombardoa* into a new synonym to *Colombiella* as the latter name takes priority. Therefore, the accepted name for the species was *Colombiella planicephala* (Rehn). Rivera (2010) synonymized the species *Margaromantis margaritaria* Piza, 1982 and *Colombiella planicephala* (Rehn, 1916) resulting in the current nomenclature of the species *Margaromantis planicephala* (Rehn).

In this paper we describe a second species of *Margaromantis* and discuss its geographic distribution.

## Materials and methods

In this study, we examined specimens collected in inventoried areas by the Programa de Pesquisa em Biodiversidade do Semiárido (PPBio / Semiárido) and specimens deposited in the Coleção Entomológica Professor Johann Becker do Museu de Zoologia da Universidade Estadual de Feira de Santana (MZFS), Feira de Santana, Bahia, Brazil. To study the male genitalia, the abdomina of the specimens were detached behind the eighth segment and treated according to the protocols of Cumming (1992). Genital nomenclature follows Cerdá (1993). The specimens studied were deposited in MZFS.

The program QGIS 2.0.1 – Dufour was used to construct the map.

## Taxon treatments

### Margaromantis nigrolineata sp. n.

urn:lsid:zoobank.org:act:D4501376-9039-4E5C-9DF9-4A2693F077DF

#### Materials

- **Type status:** Holotype. **Occurrence:** recordNumber: MZFS #13.257; recordedBy: Bravo, F.; individualCount: 1; sex: male; lifeStage: adult; **Location:** continent: South America; country: Brazil; countryCode: BRA; stateProvince: Bahia; municipality: Mucugê; verbatimElevation: 950 m; verbatimLatitude: 13°17'22.6"S; verbatimLongitude: 41°53'19.9"W; **Event:** eventDate: 2002-11-09; **Record Level:** institutionCode: UEFS; collectionCode: MZFS
- **Type status:** Paratype. **Occurrence:** recordNumber: MZFS #54.894; recordedBy: Bravo, F., Carvalho, J. R., Cordeiro, D., Menezes, E., Nascimento, F. E.; individualCount: 1; sex: male; lifeStage: adult; **Location:** continent: South America; country: Brazil; countryCode: BRA; stateProvince: Bahia; municipality: Abaíra; verbatimLocality: Catolés, Catolés de Cima, Cachoeira do Pinga-Pinga; verbatimElevation: 1219 m; verbatimLatitude: 13°17'22.6"S; verbatimLongitude: 41°53'19.9"W; **Event:** samplingProtocol: light trap; eventDate: 2013-11-03; **Record Level:** institutionCode: UEFS; collectionCode: MZFS
- **Type status:** Paratype. **Occurrence:** recordNumber: MZFS #54.895; recordedBy: Menezes, E., Nascimento, F.E., Silva-Neto, A.; individualCount: 1; sex: male; lifeStage: adult; **Location:** continent: South America; country: Brazil; countryCode: BRA; stateProvince: Bahia; municipality: Maracás; verbatimLocality: Fazenda Bom Futuro; verbatimElevation: 935 m; verbatimLatitude: 13°28'29"S; verbatimLongitude: 40°26'30"W; **Event:** samplingProtocol: light trap; eventDate: 2012-03-22/23; **Record Level:** institutionCode: UEFS; collectionCode: MZFS
- **Type status:** Paratype. **Occurrence:** recordNumber: MZFS #45.929; recordedBy: Alvim, E., Mota, E., Silva-Neto, A., Zacca,T.; individualCount: 1; sex: male; lifeStage: adult; **Location:** continent: South America; country: Brazil; countryCode: BRA; stateProvince: Bahia; municipality: Morro do Chapéu; verbatimLocality: Capão do Pinho; verbatimLatitude: 11°36'S; verbatimLongitude: 41°01'W; **Event:** eventDate: 2008-09-29/30; **Record Level:** institutionCode: UEFS; collectionCode: MZFS
- **Type status:** Paratype. **Occurrence:** recordNumber: MZFS #52.410; recordedBy: Bravo, F., Zacca, T., Silva-Neto, A., Resende, J., Almeida; individualCount: 1; sex: male; lifeStage: adult; **Location:** continent: South America; country: Brazil; countryCode: BRA; stateProvince: Bahia; municipality: Palmeiras; verbatimLocality: Posto do Pai Inácio; verbatimElevation: ca. 900 m; verbatimLatitude: 12°27'S; verbatimLongitude: 41°28'W; **Event:** samplingProtocol: light trap; eventDate: 2007-12-09; **Record Level:** institutionCode: UEFS; collectionCode: MZFS
- **Type status:** Paratype. **Occurrence:** recordNumber: MZFS #52.411; recordedBy: Bravo, F., Zacca, T., Silva-Neto, A., Resende, J., Almeida; individualCount: 1; sex: male; lifeStage: adult; **Location:** continent: South America; country: Brazil; countryCode: BRA; stateProvince: Bahia; municipality: Palmeiras; verbatimLocality: Posto do Pai Inácio; verbatimLatitude: 12°27'S; verbatimLongitude: 41°28'W; **Event:** samplingProtocol: light trap; eventDate: 2007-12-09; **Record Level:** institutionCode: UEFS; collectionCode: MZFS
- **Type status:** Paratype. **Occurrence:** recordNumber: MZFS #52.412; recordedBy: Bravo, F., Zacca, T., Silva-Neto, A., Resende, J., Almeida; individualCount: 1; sex: male; lifeStage: adult; **Location:** continent: South America; country: Brazil; countryCode: BRA; stateProvince: Bahia; municipality: Palmeiras; verbatimLocality: Posto do Pai Inácio; verbatimLatitude: 12°27'S; verbatimLongitude: 41°28'W; **Event:** samplingProtocol: light trap; eventDate: 2007-12-09; **Record Level:** institutionCode: UEFS; collectionCode: MZFS
- **Type status:** Paratype. **Occurrence:** recordNumber: MZFS #54.866; recordedBy: Silva-Neto, A., Zacca, T.; individualCount: 1; sex: male; lifeStage: adult; **Location:** continent: South America; country: Brazil; countryCode: BRA; stateProvince: Bahia; municipality: Palmeiras; verbatimLatitude: 12°27'42.08"S; verbatimLongitude: 41°28'13.00"W; **Event:** samplingProtocol: active collection; eventDate: 2007-12-08; **Record Level:** institutionCode: UEFS; collectionCode: MZFS
- **Type status:** Paratype. **Occurrence:** recordNumber: MZFS #45.910; recordedBy: Lopes, P., Menezes, E., Mota, E., Zacca, T.; individualCount: 1; sex: male; lifeStage: adult; **Location:** continent: South America; country: Brazil; countryCode: BRA; stateProvince: Bahia; municipality: Senhor do Bonfim; verbatimLocality: Serra da Maravilha; verbatimLatitude: 10°26'31.15"S; verbatimLongitude: 40°13'35.95"W; **Event:** samplingProtocol: light trap; eventDate: 2009-07-20/21; **Record Level:** institutionCode: UEFS; collectionCode: MZFS

#### Description

Male: Body (Fig. 1a) stout, green in color; length from eyes to subgenital plate, 35.55 mm.

Head (Fig. 1b) triangular in shape, ca. 1.73 times wider than pronotum. Antenna green, filiform, ca. 2.68 times longer than pronotum. Compound eyes rounded. Ocelli elliptical. Vertex exhibiting transverse black strip between the compound eyes. Frontal shield: width ca. 1.5 times greater than length, with upper medial angle acute.

Thorax (Fig. 1c). Pronotum: ca. 0.22 times as long as body; lateral margin smooth. Prozona ca. 0.37 times as long as pronotum, anterior margin rounded, lateral margins parallel. Metazona ca. 1.69 times longer than prozona. Supracoxal dilatation poorly developed. Supracoxal fissure conspicuous. Medial carina lightly projected on anterior region, absent on posterior region.

Fore coxae stout, surpasing the base of proesternum, ca. 0.80 times as long as pronotum; anterior margin with spaced ivory spines; inner face with minute tubercles.

Forefemora: stout, triangular, ca. 1.07 times longer than pronotum; 6 external spines, 14/14 inner spines (paratypes: 12/14 MZFS #54.894; 13/13 MZFS #52412; 14/13 MZFS #54.895) and 4 discoidal spines; spines of the three series black at tip. Inner face of the fore femur exhibiting a longitudinal series of seven circular black callouses, two of them occurring before the groove and the other five calluses beyond it (four of them near to the base of the largest internal spine) (Fig. 1d). Fore tibia ca. 0.63 times as long as pronotum (apical tibial claw not included) with 19/15 external spines (paratypes: 18/17 MZFS #54.894; 18/18 MZFS #45.929; 16/17 MZFS #45.910; 20/19 MZFS #52.410; 19/21 MZFS #52.411; 17/19 MZFS #52.412; 19/20 MZFS #54.866; 21/21 MZFS #54.895), 14/14 inner spines (paratypes: 15/14 MZFS #54.894; 14/15 MZFS #45.929, MZFS #52.411 and MZFS #54.866; 13/14 MZFS #45.910; 14/13 MZFS #52.410 and MZFS #52.412); spines of the two series black at tip.

Mesothoracic wings: ca. 4.17 times longer than pronotum and long as the metathoracic wings when folded, wings extend beyond the tip of abdomen. Veins green. Venules of the costal area anastomosed in basal 1/5, straight and parallel in upper 4/5.

Metathoracic wings: ca. 3.63 times longer than pronotum; wing membrane hyaline with light green apex above the anterior medial vein; yellowish strips over transversal veins absent (Fig. 1a). Veins green.

Mid and hind legs: pilose; mid femora ca. 0.95 times as long as pronotum; mid tibia ca. 0.74 times as long as pronotum; hind femur and tibia ca. 1.06 times longer than pronotum.

Abdomen: cylindrical, ca. 2.31 times longer than pronotum. Supranal plate triangular ca. 2.00 times wider than long, distal margin rounded. Cerci pilose, cylindrical and 14 articules. Subgenital plate pilose, ovoid. Styles pilose, cylindrical.

Phallic complex

Right dorsal phallomere (Fig. 2a). Dorsal lamina triangular. Mid arm developed, with angular tip (Fig. 2b). Anterior apodeme long and narrow. Ventral plate developed, sclerotized, not projected. Ventral process sclerotized, L-shaped (Fig. 2c).

Left dorsal phallomere (Fig. 2d). Triangular in shape. Dorsal lamina wide. Ventral lamina rectangular, narrow and long. Lateral process elongated and grooved, derived from right base of the ventral lamina. Apical process flattened, not twisted, upwardly recurved (Fig. 2e).

Ventral phallomere (Fig. 2f). Elliptical in shape. Distal margin straight. Distal margin and right margin more sclerotized than medial portion of the phallomere. Lateral process short, arcuated rightward, little sclerotized.

Female. Unknown. Probably the female has the same pattern of male color, with black calluses on forefemora and black strip on the vertex.

##### Measurements

Body length: holotype 35.55 mm (paratypes: 31.81–37.72 mm); head width: 5.69 mm (5.09–6.03 mm); pronotum length: 7.82 mm (7.00–8.30 mm); fore coxae: 6.26 mm (5.60–6.29 mm); fore femora: 8.39 mm (7.50–8.90 mm); fore tibia: 4.93 mm (4.41–5.23 mm); mesothoracic wings: 32.62 mm (29.20–34.63 mm); mid femura: 7.45 mm (6.67–7.91 mm); mid tibia: 5.83 mm (5.22–6.19 mm); metathoracic wings: 28.45 mm (25.46–30.20 mm); hind femura and hind tibia: 8.33 mm (7.45–8.84 mm); abdomen: 18.08 mm (16.18–19.19 mm).

#### Diagnosis

Vertex with a transverse black strip between compound eyes. Fore femora exhibiting black calluses on inner face. Metathoracic wings lacking yellowish strips over transverse veins. Left dorsal phallomere exhibiting a rectangular ventral lamina; lateral process elongated, grooved; apical process flattened and not twisted, upwardly recurved.

#### Etymology

The species epithet *nigrolineata* refers to the transverse black strip present on the vertex.

#### Distribution

**Figure 3.**
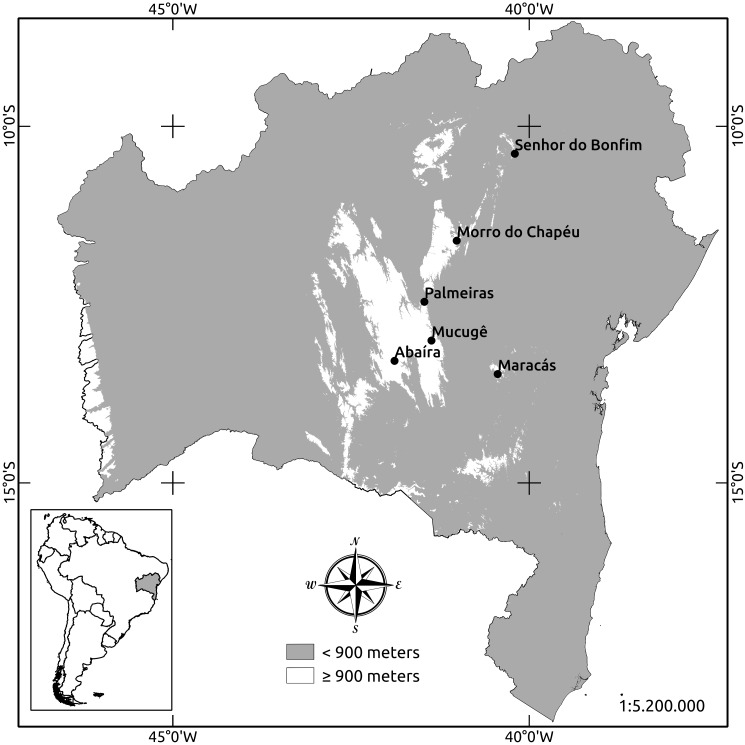
Geographical records of *Margaromantis nigrolineata* sp. n. in the state of Bahia.

*Margaromantis nigrolineata* sp. n. is currently found at an altitude above 900 m in areas of central Bahia with a semiarid climate (Fig. 3). Except for the locality of Maracás, all records for the new species are in the Chapada Diamantina mountains (Mucugê, Catolés, Morro do Chapéu, Palmeiras and Senhor do Bonfim). Maracás is located approximately 65 km east of the Chapada Diamantina mountains.

The Chapada Diamantina mountains are in the northern area of the Espinhaço Range, which extends from the state of Minas Gerais to Bahia (Rocha et al. 2005). With support from the Programa de Pesquisa em Biodiversidade do Semiárido (PPBio/Semiárido), entomological collection trips were also carried out west, east and north of Chapada Diamantina and in remnants of the Atlantic Rain Forest in the state of Bahia. However specimens of *Margaromantis nigrolineata* sp. n. were not found on these trips. These results suggest that this new species may be endemic to the mountainous areas above 900 m altitude in central Bahia. Considering that specimens were not collected in the meridian part of Chapada Diamantina mountains beyond the Abaíra, it is possible that this species also occurs south of the Espinhanço Range.

#### Taxon discussion

*Margaromantis nigrolineata* sp. n. differs from *M. planicephala* by the following characteristics: 1) presence of a black strip in the vertex (Fig. 1b), absent in *M. planicephala*; 2) fore femora with circular black callouses on the inner face (Fig. 1d), whereas in *M. planicephala* they are ivory; 3) membrane of the metathoracic wings completely hyaline (Fig. 1a), without the yellowish transverse strips present in *M. planicephala*; 4) the dorsal lamina in the left dorsal phallomere wider (Fig. 2d) than in *M. planicephala*; 5) ventral lamina in the left dorsal phallomere transversely oriented, rectangular and developed, reaching the anterior margin in *M. nigrolineata* sp. n. (Fig. 2e) and oblique, reduced and restricted the posterior region in *M. planicephala*; 6) apical process in the left dorsal phallomere directed upward and not twisted in the new species (Fig. 2e), whereas directed sinistrally and twisted in *M. planicephala*; 7) right margin of ventral phallomere a little more sclerotized in the new species than in *M. planicephala*.

## Supplementary Material

XML Treatment for Margaromantis nigrolineata

## Figures and Tables

**Figure 1a.**
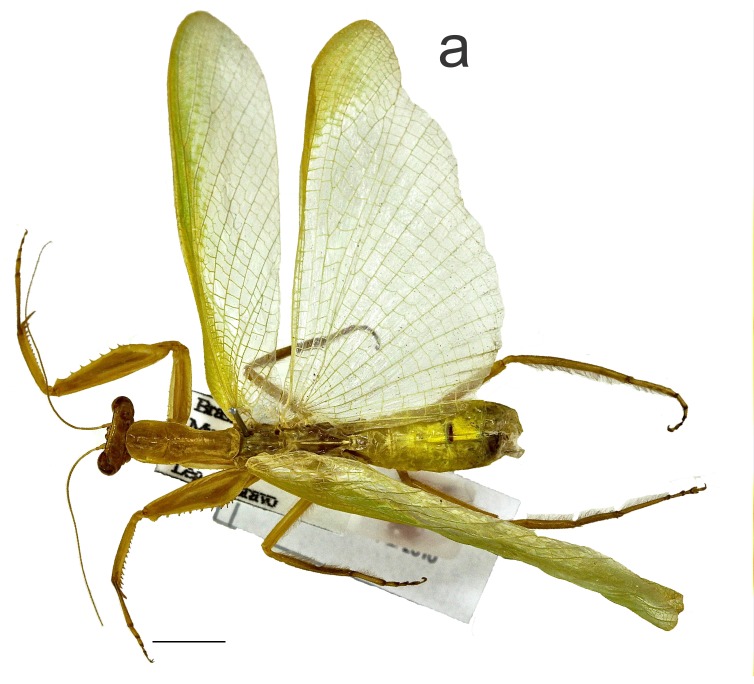
Dorsal habitus, scale bar = 5.00 mm.

**Figure 1b.**
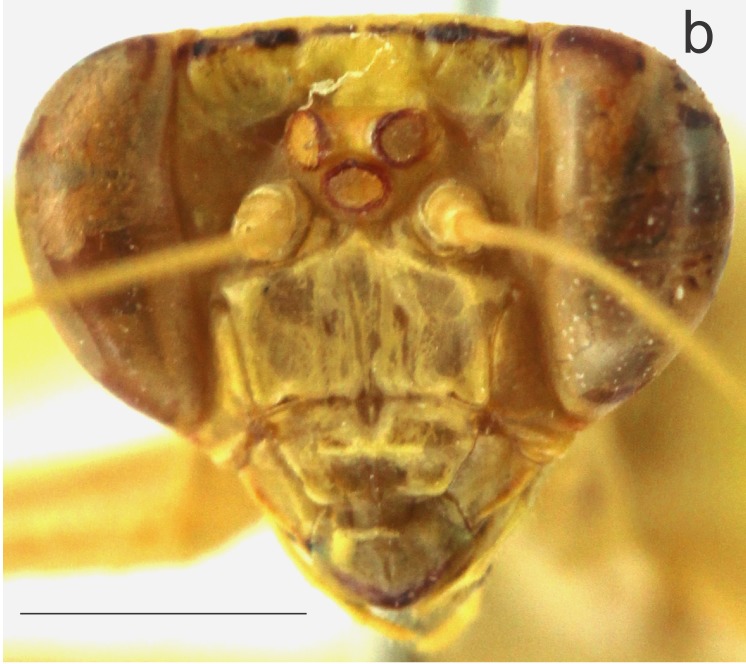
Head, frontal view, scale bar = 2.00 mm.

**Figure 1c.**
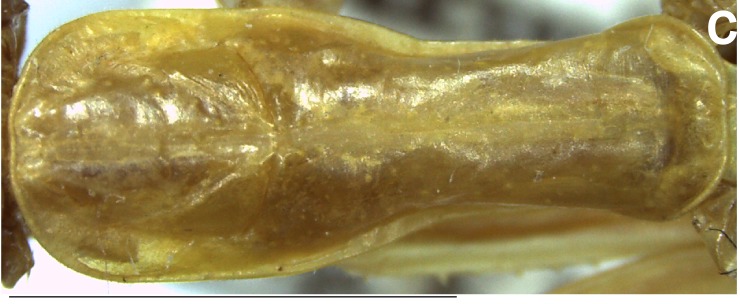
Pronotum, dorsal view, scale bar = 5.00 mm.

**Figure 1d.**
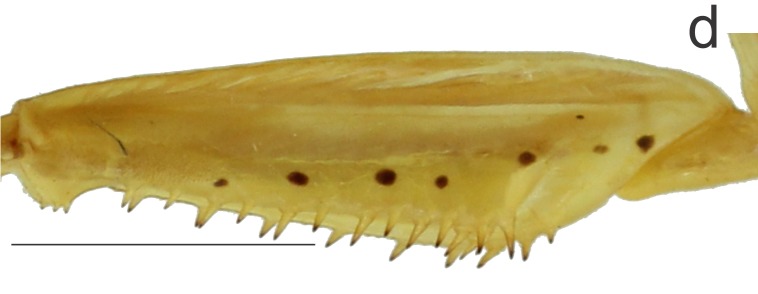
Right fore femur, inner view, scale bar = 5.00 mm.

**Figure 2a.**
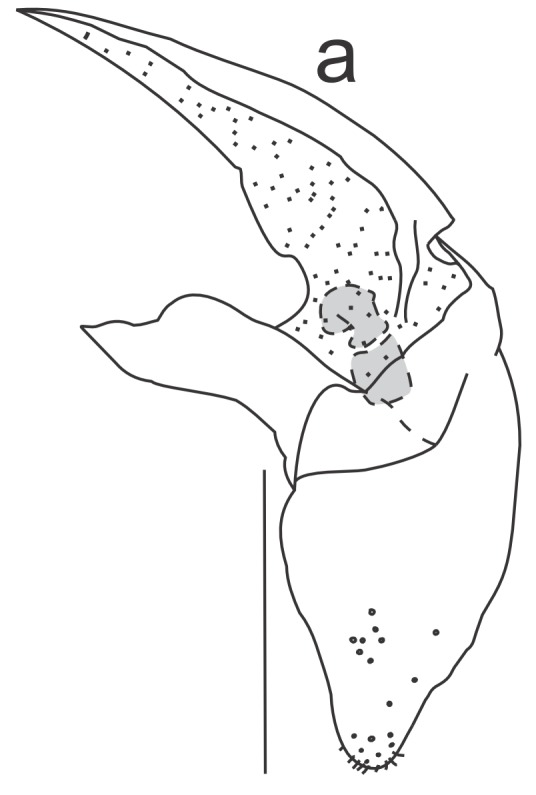
Dorsal right phallomere, dorsal view.

**Figure 2b.**
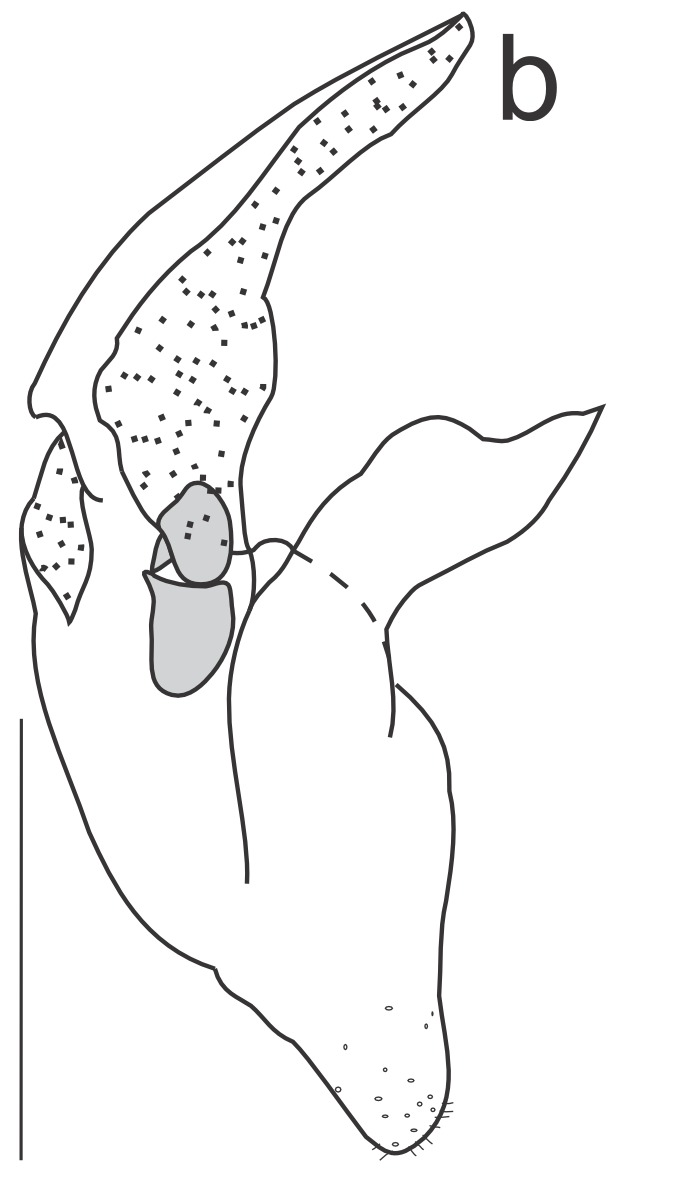
Dorsal right phallomere, ventral view.

**Figure 2c.**
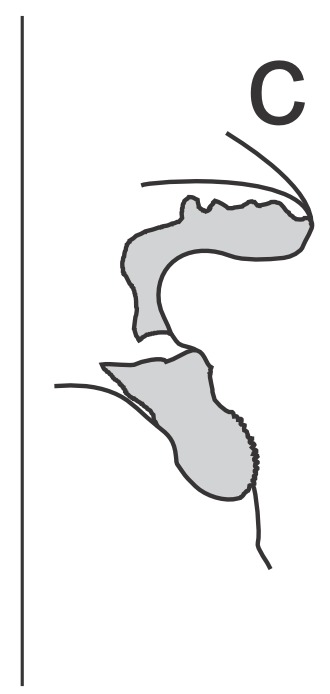
Ventral process and ventral plate, lateral view.

**Figure 2d.**
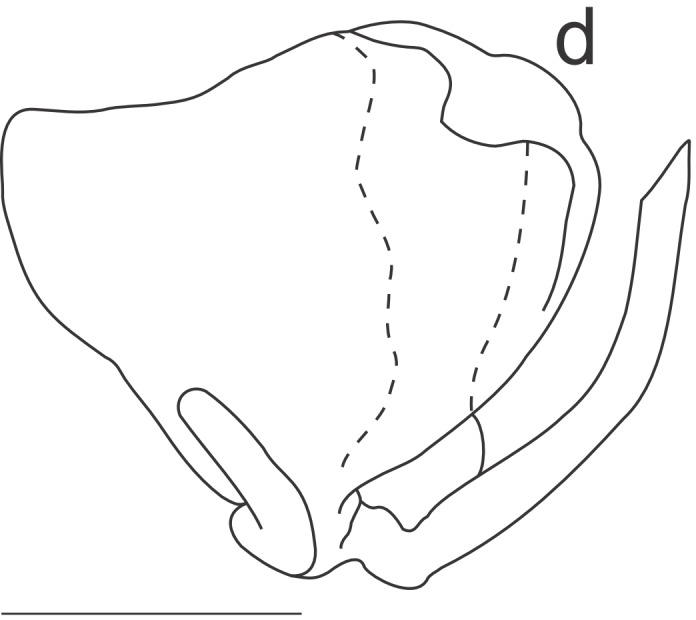
Dorsal left phallomere, dorsal view.

**Figure 2e.**
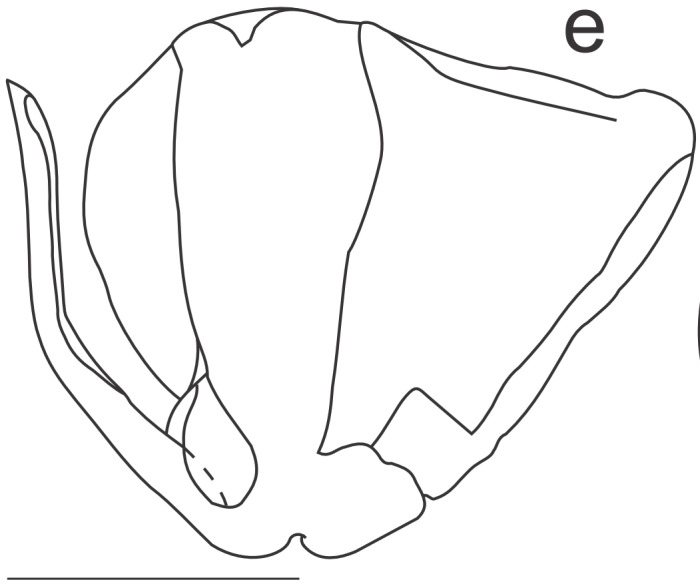
Dorsal left phallomere, ventral view.

**Figure 2f.**
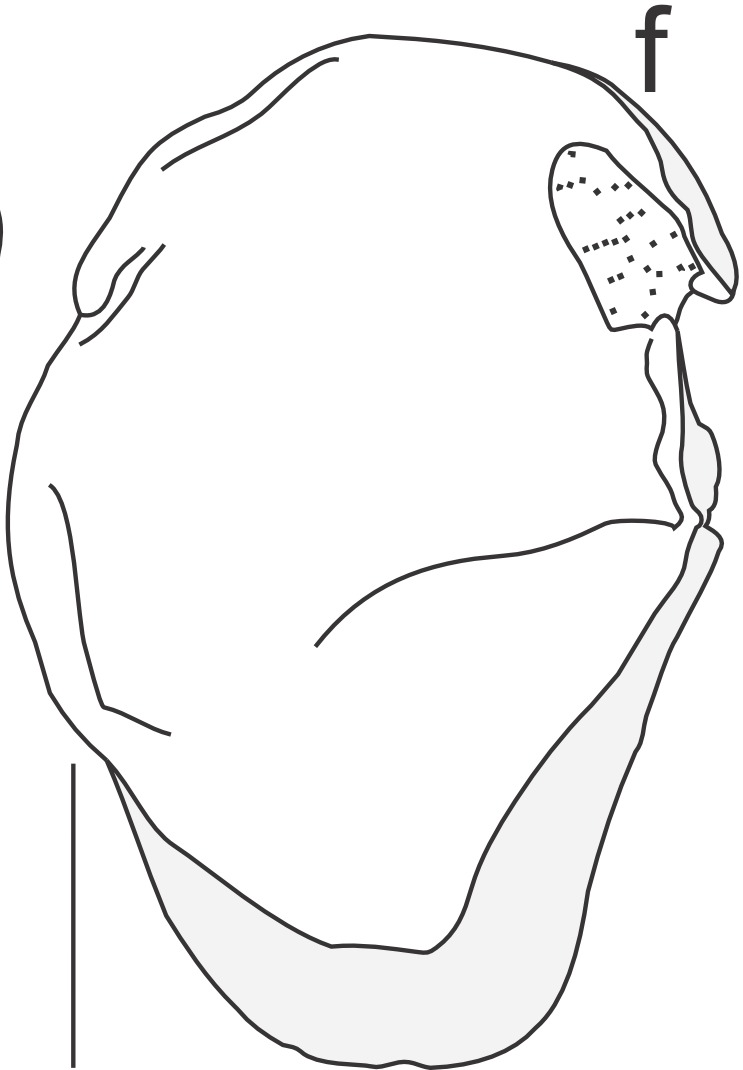
Ventral phallomere, dorsal view. All scale bars = 1.00 mm.
